# Are 5-level triage systems improved by using a symptom based approach?—a Danish cohort study

**DOI:** 10.1186/s13049-022-01016-2

**Published:** 2022-04-25

**Authors:** Frederik Trier Kongensgaard, Marianne Fløjstrup, Annmarie Lassen, Jan Dahlin, Mikkel Brabrand

**Affiliations:** 1 Department of Emergency Medicine, Hospital of South West Jutland, Esbjerg, Denmark; 2 Department of Regional Health Research, University of Southern Denmark, Odense, Denmark; 3 Department of Clinical Research, University of Southern Denmark, Odense, Denmark; 4 Department of Emergency Medicine, Odense University Hospital, Odense, Denmark

**Keywords:** Triage systems, Triage, Symptoms, Vital signs, Chief complaint, Mortality, ICU, Surgery, ED

## Abstract

**Background:**

Five-level triage systems are being utilized in Danish emergency departments with and without the use of presenting symptoms. The aim of this study was to validate and compare two 5-level triage systems used in Danish emergency departments: “Danish Emergency Process Triage” (DEPT) based on a combination of vital signs and presenting symptoms and a locally adapted version of DEPT (VITAL-TRIAGE) using vital signs only.

**Methods:**

This was a retrospective cohort using data from five Danish emergency departments. All patients attending an emergency department during the period of 1 April 2012 until 31 December 2015 were included. Validity of the two triage systems was assessed by comparing urgency categories determined by each triage system with critical outcomes: admission to Intensive care unit (ICU) within 24 h, 2-day mortality, diagnosis of critical illness, surgery within 48 h, discharge within 4 h and length of hospital stay.

**Results:**

We included 632,196 ED contacts. Sensitivity for 24-h ICU admission was 0.79 (95% confidence interval 0.78–0.80) for DEPT and 0.44 (0.41–0.47) for VITAL-TRIAGE. The sensitivity for 2-day mortality was 0.69 (0.67–0.70) for DEPT and 0.37 (0.34–0.41) for VITAL-TRIAGE. The sensitivity to detect diagnoses of critical illness was 0.48 (0.47–0.50) for DEPT and 0.09 (0.08–0.10) for VITAL-TRIAGE. The sensitivity for predicting surgery within 48 h was 0.30 (0.30–0.31) in DEPT and 0.04 (0.04–0.04) in VITAL-TRIAGE. Length of stay was longer in VITAL-TRIAGE than DEPT. The sensitivity of DEPT to predict patients discharged within 4 h was 0.91 (0.91–0.92) while VITAL-TRIAGE was higher at 0.99 (0.99–0.99). The odds ratio for 24-h ICU admission and 2-day mortality was increased in high-urgency categories of both triage systems compared to low-urgency categories.

**Conclusions:**

High urgency categories in both triage systems are correlated with adverse outcomes. The inclusion of presenting symptoms in a modern 5-level triage system led to significantly higher sensitivity measures for the ability to predict outcomes related to patient urgency. DEPT achieves equal prognostic performance as other widespread 5-level triage systems.

**Supplementary Information:**

The online version contains supplementary material available at 10.1186/s13049-022-01016-2.

## Introduction

Triage systems are essential in a modern emergency department (ED). More than a million patients are referred to and seen in Danish EDs each year [1]. The increasing number of patients can result in crowding and prolonged waiting time when the amount of available healthcare resources are exceeded. Triage systems have been implemented to prioritize patients with the purpose of identifying high-risk patients that require immediate medical attention and to safely determine who will not be disadvantaged by longer waiting times [2].

In Denmark, most EDs have implemented formalized triage with a 5-level triage system called “Danish Emergency Process Triage” (DEPT) [3, 4]. DEPT shares core similarities with widespread standardized 5-level triage systems such as Manchester Triage System (MTS), Australian Triage Scale (ATS) and Canadian Emergency Department Triage and Acuity Scale (CTAS) [5]. Standardized triage systems rely on the ABCDE principles and consist of five colour-coded categories based on vital signs and presenting symptoms [5]. The categories determine how fast a patient should be assessed by a physician upon arrival to the ED.

Patients who require immediate intervention are often referred to as “urgent”, however, there is no established consensus on what determines true “urgency” [6]. In the validation process of triage systems, the most commonly used method to measure true urgency is through “construct validity” where selected indicators of a critical hospitalization are chosen and compared to triage category [6].

Modern 5-level triage systems are based on expert opinion and there is a general agreement that relevant multicentre studies on ED triage are lacking [5, 7]. In a recent review, triage systems were reported to have reasonable to good performance on the ability to identify high urgency patients based on admission to Intensive care unit (ICU) but a general weakness in identifying patients with critical illness outcomes and mortality during hospitalization [5].

The use of presenting symptoms has been an integrated part of triage systems for decades [8]. However, there is an absence of studies examining the influence of presenting symptoms on the ability of a triage system to predict adverse outcomes [9]. To our knowledge, no existing literature has examined the differences between a triage system using both vital signs and presenting symptoms, and a triage system utilizing vital signs only.

### Objectives

The aim of this study was to validate and compare two triage systems used in Danish emergency departments. A triage system based on vital signs and presenting symptoms (DEPT) and a triage system using vital signs only (VITAL-TRIAGE). The validation relies on the predictive performance of each system by comparing triage categories with predetermined outcomes that reflect a critical or non-critical hospitalization.

## Methods

We reported this study based on The Strengthening the Reporting of Observational Studies in Epidemiology (STROBE) statement and the STROBE explanation and elaboration [10, 11].

### Study design

This is a retrospective cohort study based on a secondary analysis of data from “Syddanske Akutkohorte” (SAK-cohort) [12]. The cohort includes all adults who were seen in an emergency department in the Region of Southern Denmark from 1 April 2012 until 31 December 2015. Patients under 18 years and patients without a Danish personal identification number were excluded. We included all ED contacts despite individual patients having multiple visits. All patients were followed until discharge or death whichever came first.

### Setting

The SAK-cohort included data from five EDs: Odense University Hospital (OUH), Odense University Hospital Department at Svendborg (OUHS), Hospital of South Jutland (SHS), Hospital of Lillebaelt (SLB) and Hospital of South West Jutland (SVS).

OUH is a university hospital and Level I trauma centre. Together with OUHS, they manage approximately 100,000 patients yearly [13]. SHS, SLB and SVS are regional teaching hospitals. They handle approximately 32,000 (SHS), 33,000 (SVS) and 50,000 (SLB) patients yearly [14–16] (Table 1). All regional hospitals offer 24-h emergency care and level-2 trauma including broad medical, surgical, neurological and ICU services.

**Table 1:** Baseline data on involving centers [13–16]

	DEPT			VITAL-TRIAGE
Location	OUH + OUHS	SHS	SLB	SVS
Regional uptake	430,000	235,000	300,000	253,000
Hospital beds	1038	367	568	370
Discharged patients per year	104,000	32,000	48,000	33,000
Outpatient visits per year	1,130,000	430,000	516,000	390,000
No of employees	11,300	3000	5000	3000

The table containing key figures on each hospital included. Categorized by triage system

### DEPT triage

OUH, OUHS, SHS and SLB have implemented DEPT triage using vital signs and presenting symptoms since 2011 [17]. At the same time, SVS implemented a local modification of DEPT using only vital signs, VITAL-TRIAGE. The process of triage is carried out by trained staff prior to diagnostics and assessment by a physician. The triage category is based on vital signs (e.g., Glasgow Coma Scale, blood pressure, respiratory rate, heart rate, etc.) and a presenting symptom algorithm (e.g., chest pain, abdominal pain, trauma, unconsciousness etc.). The most urgent of either vital signs or presenting symptoms determines the final triage category.

In VITAL-TRIAGE, the triage category is based solely on vital signs and a visual analogue pain scale (VAS) [18] in case of surgical patients. The triage categories used in both systems are: Red (immediate evaluation by physician), Orange (emergent, evaluation within 15 min), Yellow (potentially unstable, evaluation within 60 min), Green (non-urgent, re-evaluation every 180 min), and Blue (minor injuries or complaints, re-evaluation every 240 min). In DEPT, secondary modifiers are included to increase the triage category of patients whose co-morbidity or medication might lead to an underestimation of the clinical condition at triage. Examples of secondary modifiers are existing ischemic heart disease or insulin demanding type II diabetes, primary immunodeficiencies or medication with immunosuppressants [4]. It is possible to be categorized higher than identified by DEPT and VITAL-TRIAGE if the triage nurse suspect a more critical condition, downward triage can only be done upon assessment by a physician [4]. The triage category is registered using the software package Cetrea Emergency (Getinge Cetrea A/S, Aarhus, Denmark).

### Outcome and variables

The primary outcome was admission to intensive care unit (ICU) within 24 h after ED arrival. Secondary outcomes were 2-day mortality, patients subjected to surgery within 48 h, diagnosis of critical illness, length of stay (LOS) and discharge within 4-h.

Exposure variables were each of the triage categories provided by each triages system. In addition, a Grey category was formed containing all patients who were seen in the ED but was not registered with a triage category. Individual level variables of age, gender and Charlson comorbidity index (CCI) [19] was included.

### Data sources

Information on sex, hospital, time of arrival to ED and triage category for each ED contact was retrieved from Cetrea. Cetrea data was merged with data from The Danish National Patients registry (DNPR). DNPR contributed with information on length of stay, Charlson co-morbidity index, surgical procedures, time of discharge and primary diagnosis [20]. Age was retrieved from The Danish Civil Registration System using the unique personal identification number in Cetrea [21].

For alle outcomes except critical illness, the time of registration on a Cetrea board in the ED was used as arrival time to ED. Twenty-four hour ICU admission was defined as a maximum of 24 h from arrival time to the ED until the time stamp of the first ICU admission retrieved by DNPR.

Forty-eight hour mortality was counted as death on the same day or the next day, after arrival to the ED using the date of death in The Danish Civil Registration System.

Surgery within 48 h was defined as a maximum of 48 h from arrival to the ED until time of operation as registered in DNPR.

Length of stay was measured from arrival time at ED until time of discharge as registered in DNPR, 4-h discharge was counted as an ED contact with an LOS less than 4 h.

The primary diagnosis of every ED contact was retrieved by DNPR at final hospital discharge despite the patient being submitted to hospitalization in a general ward or discharged directly from the ED.

Diagnoses of critical illness were obtained from registries of The Danish Clinical Quality Program—national clinical Registries (RKKP). RKKP asks expert clinicians to conduct clinical registries used for the improvement of quality, research and surveillance purposes [22]. We screened all databases and included those areas of critical illness where early intervention is important for optimal clinical outcomes. Four areas of disease and the corresponding ICD-10 codes were included: Stroke, Acute Coronary Syndrome, bleeding ulcer and Gastrointestinal perforation [23] (Additional file 1: Table S1).

### Statistical methods

Baseline characteristics were presented as numbers, percentages, and medians. We used $\upchi$^2^-test to compare binary outcomes. *p* values below 0.05 were considered significant and 95% confidence intervals (CI) are presented when appropriate. To describe differences in length of stay, we calculated percentiles and interquartile range. Kruskal–Wallis H test and Wilcoxon rank-sum was used to test for significant differences between triage categories and triage systems.

Odds ratios (OR) were calculated to illustrate probabilities of 2-day mortality and ICU admittance and to stratify for potential confounders.

CCI were grouped into four: 0, 1, 2 and > 2, and age into age-groups: 18–49, 50–64, 65–79 and 79+ years of age. To calculate diagnostic sensitivity and specificity we dichotomised the DEPT categories into high-urgency categories (Red and Orange) and low-urgency categories (Yellow, Green, Blue).

All analyses were conducted using STATA V 16.1 (StataCorp, Texas, USA)

### Ethics statement

The study was approved by the Danish data protection agency. Register-based studies are exempt from approval by an ethics committee according to Danish law. No further approval was required.

## Results

### Participants

A total of 632,196 ED contacts were included comprising 345,132 unique patients. We included 497,685 ED contacts in DEPT and 134,511 in VITAL-TRIAGE.

**Fig. 1:**
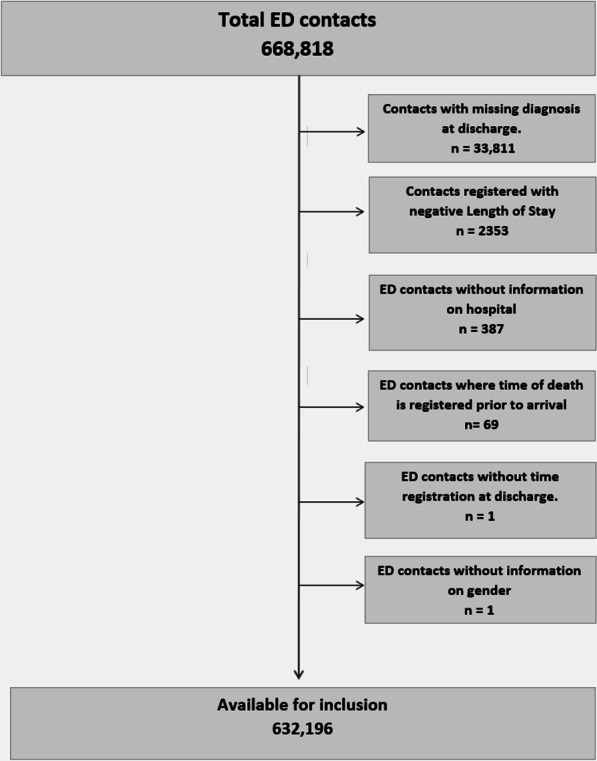
Flowchart. Flowchart describing exclusion of patients due to unreliable registration of data

We excluded 36,622 ED contacts due to unreliable registration of data (Fig. 1). In the final sample, 323,439 (51.2%) were male and the overall median age was 53 (IQR 34:72) (Table 2.)

**Table 2:** Baseline data of participants

	DEPT	VITAL-TRIAGE	*p* values
Men	253,245 (50.9%)	70,194 (52.2%)	**< 0.001**
Women	244,440 (49.1%)	64,317 (47.8%)	
Age			
Age, median (IQR)	53 (IQR 34:72)	52 (IQR 33:71)	**< 0.001**
18–49	224,888 (45.2%)	61,872 (46.0%)	
50–64	96,869 (19.5%)	26,534 (19.7%)	
65–79	103,525 (20.8%)	28,527 (21.2%)	
79+	62,403 (14.5%)	17,578 (13.1%)	
Triage categories			
Grey	108,837 (21.9%)	37,025 (27.5%)	**< 0.001**
Blue	94,601 (19.0%)	15,114 (11.2%)	
Green	117,032 (23.5%)	52,454 (39.0%)	
Yellow	103,738 (20.8%)	25,705 (19.1%)	
Orange	62,672 (12.6%)	2700 (2.0%)	
Red	10,805 (2.2%)	1513 (1.1%)	
Charlson Comorbidity Index			
CCI 0	305,568 (61.4%)	83,428 (62.0%)	**< 0.001**
CCI 1	77,421 (15.6%)	20,042 (14.9%)	
CCI 2	47,848 (9.6%)	12,673 (9.4%)	
CCI > 2	66,848 (13.4%)	18,368 (13.7)	

Bold values describe the statistical *p*-value

The table contains distributions of ED contacts into gender, age and Charlson Comorbidity Index. Horizontally categorized by triage systems

*p values were based on Pearson’s Chi-square test for categorical variables and frequencies, and Wilcoxon ranks sum test for ordinal variables

#### 24-h intensive admission

**Fig. 2:**
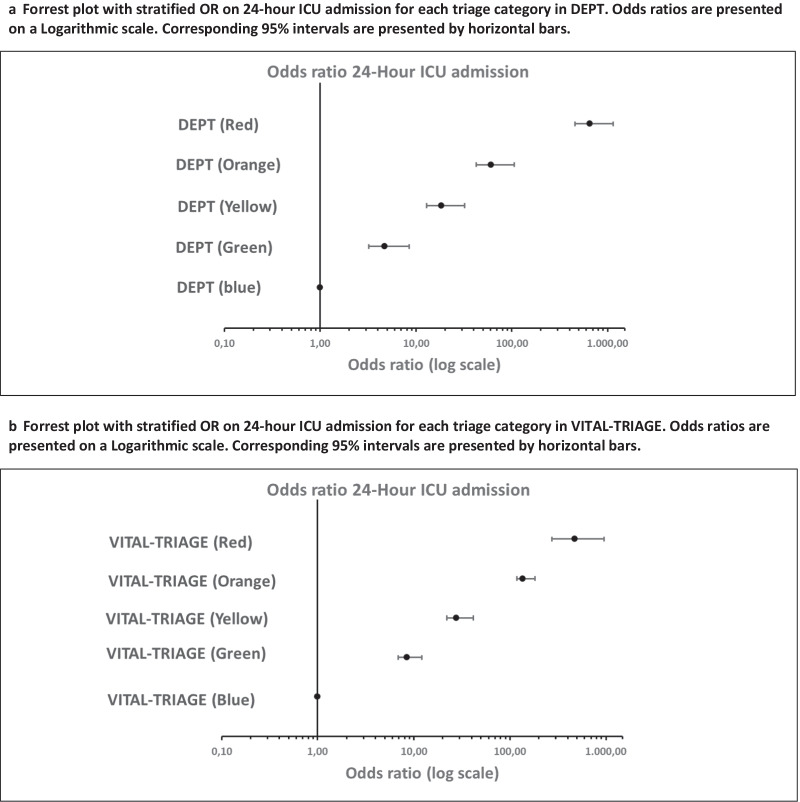
**a**, **b** Odds ratios on ICU-admission. **a** Forrest plot with stratified OR on 24-h ICU admission for each triage category in DEPT. Odds ratios are presented on a Logarithmic scale. Corresponding 95% intervals are presented by horizontal bars. **b** Forrest plot with stratified OR on 24-h ICU admission for each triage category in VITAL-TRIAGE. Odds ratios are presented on a Logarithmic scale. Corresponding 95% intervals are presented by horizontal bars

In DEPT a total of 6619 (1.3%) ED contacts were admitted into an ICU within 24 h. DEPT triaged 3993 (0.8%) into high urgency categories (Table 3). In VITAL-TRIAGE a total of 1513 (1.1%) ED contacts were admitted to an ICU and 529 (0.4%) were triaged into high-urgency categories. The sensitivity of high-urgency categories was 0.79 (CI 0.78: 0.80) in DEPT and 0.44 (CI 0.41: 0.47) in VITAL-TRIAGE. Specificity was higher in VITAL-TRIAGE (Table 4). Odds ratios for ICU admission was significantly increased in high-urgency categories relative to low-urgency categories in both systems (Additional file 1: Table S2) (Fig. 2a, b).

**Table 3:** Outcomes

Triage category	DEPT	VITAL-TRIAGE	Statistic
**24-h ICU admission**			
Grey	1582 (0.3%)	305 (0.2%)	χ^2^ *p* < 0.001
Blue	30 (0.01%)	6 (0.004%)	
Green	211 (0.04%)	238 (0.2%)	
Yellow	803 (0.2%)	435 (0.3%)	
Orange	1637 (0.3%)	210 (0.2%)	
Red	2356 (0.5%)	319 (0.2%)	
**2-day mortality**			
Grey	1691 (0.3%)	198 (0.1%)	χ^2^ *p* < 0.001
Blue	72 (0.02%)	48 (0.04%)	
Green	171 (0.04%)	162 (0.1%)	
Yellow	539 (0.1%)	237 (0.2%)	
Orange	797 (0.2%)	94 (0.07%)	
Red	974 (0.2%)	171 (0.1%)	
**48-h surgery**			
Grey	8671 (1.7%)	4799 (3.6%)	χ^2^ *p* < 0.001
Blue	4203 (0.8%)	1560 (1.2%)	
Green	10,046 (2.0%)	7722 (5.7%)	
Yellow	12,186 (2.4%)	2612 (1.9%)	
Orange	9978 (2.0%)	307 (0.2%)	
Red	1617 (0.3%)	192 (0.14%)	
**Critical illness**			
Grey	2275 (0.5%)	233 (0.2%)	χ^2^ *p* < 0.001
Blue	29 (0.01%)	17 (0.01%)	
Green	1103 (0.2%)	1606 (1.2%)	
Yellow	2395 (0.5%)	1168 (0.9%)	
Orange	2477 (0.5%)	158 (0.1%)	
Red	834 (0.2%)	115 (0.09%)	
**Length of stay (LOS)**			
Grey	3.8 (IQR 1.6: 26.4)	2.2 (IQR 0.9: 5.0)	Mann whitney test for difference between triage systems *p* < 0.05
Blue	2.1 (IQR 1.4: 3.3)	1.9 (IQR 0.8: 3.3)	
Green	3.4 (IQR 1.9: 18.4)	15.4 (IQR 3.2: 50.1)	
Yellow	17.1 ( IQR 3.3: 78.8)	31.2 (IQR 11.9: 120.1)	
Orange	25.0 (IQR 4.1: 117.2)	65.5 (IQR 16.7: 158.3)	Kruskall wallis test for difference between categories *p* < 0.05
Red	52.4 (IQR 16.1: 172.9)	43.7 (IQR 14.6: 164.5)	
**4-h discharge**			
Grey	55,444 (11.1%)	25,977 (19.3%)	χ^2^ *p* < 0.001
Blue	78,799 (15.8%)	12,463 (9.3%)	
Green	65,426 (13.1%)	15,207 (11.3%)	
Yellow	31,620 (6.4%)	2686 (2.0%)	
Orange	15,246 (3.1%)	234 (0.2%)	
Red	1134 (0.2%)	134 (0.1%)	

The table shows the number of ED contacts in each outcome. Presented by triage category vertically and the triage system horizontally. Percentage (%) is the share of all patients allocated to the triage system. Length of stay is presented as median (hours) and Interquartile Range. The result of corresponding statistical analyses is presented in the right column

**Table 4:** Performance metrics

Performance metrics	DEPT	VITAL-TRIAGE	Triage categories
**24-h ICU admission**			
Sensitivity	0.79 (CI 0.78: 0.80)	0.44 (CI 0.41: 0.47)	High-urgency categories
Specificity	0.82 (CI 0.82: 0.82)	0.96 (CI 0.96: 0.96)	
Positive predictive value	0.05 (CI 0.05: 0.06)	0.13 (CI 0.12: 0.14)	
Negative predictive value	0.99 (CI 0.99: 0.99)	0.99 (CI 0.99: 0.99)	
**2-day mortality**			
Sensitivity	0.69 (CI 0.67: 0.70)	0.37 (CI 0.34: 0.41)	High-urgency categories
Specificity	0.81 (CI 0.81: 0.82)	0.96 (CI 0.96: 0.96)	
Positive predictive value	0.02 (CI 0.02: 0.03)	0.06 (CI 0.06: 0.07)	
Negative predictive value	0.99 (CI 0.99: 0.99)	0.99 (CI 0.99: 0.99)	
**Critical illness**			
Sensitivity	0.48 (CI 0.47: 0.50)	0.09 (0.08: 0.10)	High-urgency categories
Specificity	0.85 (CI: 0.84: 0.85)	0.96 (CI 0.96: 0.96)	
Positive predictive value	0.05 (CI 0.04: 0.05)	0.06 (CI 0.06: 0.07)	
Negative predictive value	0.99 (CI 0.99: 0.99)	0.97 (CI 0.97: 0.97)	
**Surgery within 48 h**			
Sensitivity	0.30 (CI 0.30: 0.31)	0.04 (CI 0.04: 0.04)	High-urgency categories
Specificity	0.82 (CI 0.82: 0.82)	0.96 (CI 0.95: 0.96)	
Positive predictive value	0.16 (CI 0.16: 0.16)	0.12 (CI 0.11: 0.13)	
Negative predictive value	0.92 (CI 0.92: 0.92)	0.87 (CI 0.87: 0.87)	
**4-h discharge**			
Sensitivity	0.91 (CI 0.91: 0.92)	0.99 (CI 0.99: 0.99)	Low-urgency categories
Specificity	0.29 (CI 0.29: 0.29)	0.06 (CI 0.06: 0.06)	
Positive predictive value	0.56 (CI 0.56: 0.56)	0.33 (CI 0.32: 0.33)	
Negative predictive value	0.78 (CI 0.77: 0.78)	0.91 (CI 0.90: 0.92)	

Table of performance metrics for each triage system. Corresponding 95% confidence intervals are presented. The triage categories examined are shown in the right column

#### Two-day mortality

**Fig. 3:**
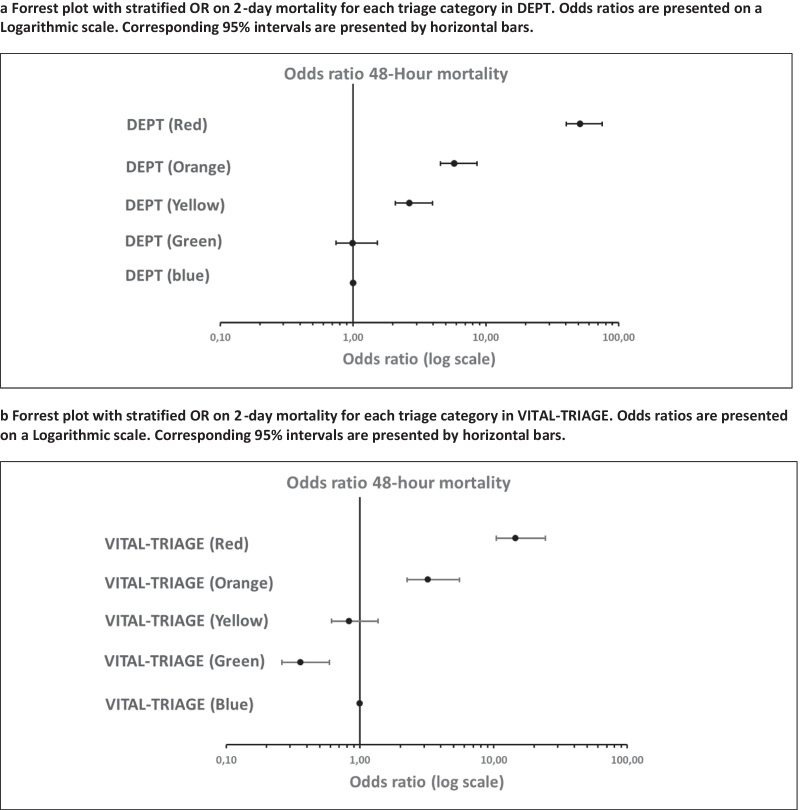
**a**, **b** Odds ratios on 2-day mortality. **a** Forrest plot with stratified OR on 2-day mortality for each triage category in DEPT. Odds ratios are presented on a Logarithmic scale. Corresponding 95% intervals are presented by horizontal bars. **b** Forrest plot with stratified OR on 2-day mortality for each triage category in VITAL-TRIAGE. Odds ratios are presented on a Logarithmic scale. Corresponding 95% intervals are presented by horizontal bars

In DEPT a total of 4244 (0.9%) patients died within 2 days of ED arrival. DEPT triaged 1771 (0.4%) into high urgency categories and 782 (0.2%) into low urgency categories (Table 3). In VITAL-TRIAGE a total of 910 (0.5%) patients died within 2 days of ED arrival, of these 265 (0.2%) patients were triaged into high-urgency categories and 447 (0.3%) into low-urgency categories. The sensitivity of detecting 2-day mortality in high urgency categories was 0.69 (CI 0.67: 0.70) in DEPT which was significantly higher than 0.37 (CI 0.34: 0.41) in VITAL-TRIAGE (Table 4). Specificity was higher in VITAL-TRIAGE. Odds ratios for 2-day mortality was significantly higher in high-urgency categories relative to low-urgency categories in both systems (Additional file 1: Table S3) (Fig. 3a, b).

#### Critical illness

In DEPT we registered 9113 (1.8%) ED contacts with a critical illness diagnosis at final discharge. DEPT triaged 3311 (0.7%) of these into high urgency categories (Table 3).

In VITAL-TRIAGE 3297 (2.5%) ED contacts were discharged with a critical illness. VITAL-TRIAGE triaged only 273 (0.2%) into high urgency categories (Table 3). The sensitivity of DEPT was higher at 0.48 (CI 0.47: 0.50) compared to 0.09 (CI 0.08: 0.10) for VITAL-TRIAGE. Specificity was higher in VITAL-TRIAGE (Table 4).

#### Surgery within 48 h

In DEPT a total of 46,701 (9.4%) ED contacts was subjected to a surgery within 48 h. DEPT triaged 11,595 (2.4%) into high-urgency categories and 26,435 (0.4%) into low-urgency categories (Table 3). In VITAL-TRIAGE 17,192 (12.8%) ED contacts were subjected to a surgery. VITAL-TRIAGE categorized 499 (0.4%) into high-urgency categories and 11,894 (12.8%) into low-urgency categories. DEPT had a sensitivity of 0.30 (CI 0.30: 0.31) while VITAL-TRIAGE were remarkably lower at 0.04 (CI 0.04: 0.04) (Table 4).

#### Length of stay

We found LOS to be significantly different between triage categories in both triage systems. VITAL-TRIAGE showed longer LOS across all triage categories except Blue (Table 3).

The percentage of patients discharged within 4 h in high-urgency categories were more than twice as high in DEPT and almost a fourth of all Orange patients were discharged within 4 h in DEPT.

VITAL-TRIAGE reached the highest sensitivity of 0.99 (CI: 0.99: 0.99) on the ability to triage 4-h discharge patients into low-urgency categories (Table 4).

#### Grey patients

Grey patients had higher stratified OR for ICU admittance within 24 h in both triage systems (Additional file 1: Table S2). The OR for 2-day mortality was significantly increased in DEPT while the OR was indifferent to blue category in VITAL-Triage (Additional file 1: Table S3).

## Discussion

In this retrospective cohort study, we validated and compared two triage systems utilized in Danish EDs. A modern 5-level triage system based on presenting symptoms and vital signs, DEPT, and a locally adapted triage system VITAL-TRIAGE based on vital signs only. We found that a triage system utilizing presenting symptoms performed significantly better at predicting 24-h intensive admission, 2-day mortality, need for surgery and critical illness.

The results of this study show that the inclusion of presenting symptoms at triage leads to increased allocation of patients with urgent conditions into high-urgency categories but at the expense of systematic over-triage of patients with non-urgent conditions.

Several studies have investigated the prognostic value of presenting symptoms at ED arrival, but the effect of a symptom based approach in the triage process is less known [24–27]. A former study on a DEPT predecessor found that in the triage process, vital signs are superior to presenting symptoms at predicting adverse outcomes [24]. They also conclude that the inclusion of presenting complaints in the triage process may lead to over-triage. Our study suggests that substantial prognostic value is lost when excluding presenting symptoms in a triage system.

While ICU admission and mortality are frequently used outcomes in the validation of triage systems, consensus on a safe level of sensitivity and specificity is not established [28]. We find that the high-urgency categories of both triage systems are correlated with increased risk of ICU admission and mortality. DEPT showed significantly higher levels of sensitivity in both outcomes. DEPT achieves similar performance in predicting ICU admission as other standardized 5-level triage systems [7].

Modern 5-level triage systems have been shown to categorize less than 80% of patients who die after emergency care into high-urgency categories [5]. In this study, DEPT triages 69% of patients who die within 2-days of ED arrival into high-urgency categories, whereas VITAL-TRIAGE detects 37%. Some of these patients are expected due to the referral of terminal and palliative patients to the ED. The allocation of these groups into low-urgency groups is not specified in DEPT or VITAL-TRIAGE [4]. We are not aware of any studies describing the triage of these patients or how they contribute to estimations of 2-day mortality. Odds ratios of mortality were associated with triage categories in DEPT. In VITAL-TRIAGE we found an unexpected increased OR of Blue category compared with Green and Yellow categories. We are not aware of the specific reasons behind this association. It is a possibility that incorrect triage of patients with high-risk conditions can lead to delayed assessment and increased mortality.

To examine the triage of critical illnesses we pooled four subgroups of conditions for which early intervention is important to reach optimal outcomes. The exclusion of presenting symptoms led to higher specificity in VITAL-TRIAGE but significantly lower sensitivity than DEPT. The data indicate that a disproportionate number of patients with a critical illness are overlooked when not accounted for the presenting symptom in triage. While the presenting symptoms appear to be an important indicator of an acute illness, DEPT is unable to detect more than half of the patients discharged with a diagnosis of critical illness as high-urgency patients. Similarly, a previous study on ADAPT found that it could not identify patients with gastro intestinal perforation [29].

Increased LOS and urgency of triage levels showed significant correlation within both triage systems. Several reviews on other 5-level triage systems find the same association between triage category and LOS [30, 31]. LOS was generally longer in VITAL-TRIAGE. It could be assumed that longer LOS happen as a consequence of a triage system’s inability to limit ED crowding, however, it has recently been emphasized that LOS depends highly on ED characteristics rather than an effect of the implemented triage system [7, 32].

We introduce discharge within 4-h as a measure of a triage system’s ability to limit crowding, the results show that the inclusion of presenting symptoms in triage lead to lower sensitivity and over-triage.

## Strength and limitations

The purpose of this study was two-fold, we wanted to validate a modern 5-level triage system and to investigate the effect of presenting symptoms in triage. The strength of this study relies on the large and unselected cohort of patients included. We examined the baseline data of patients included in each triage system. Statistical test of association suggest significant differences, however this was expected due to the large inclusion. We included both medical and surgical patients in our cohort. The cohort did not include pediatric and adolescents which is a limitation of our study.

Using data from DNPR we achieved complete follow up of all ED contacts.

When using 2-day mortality as an outcome, it is suspected that patients admitted to palliative care are contributing to an overestimation in low-urgency categories. Given the size of this cohort, it is not an option to examine the medical records and identify these patients. Due to the date of death being retrieved from DNPR and not linked to the specific hospital course, the mortality count is potentially overestimated due to patients who had more than one ED contact in the 2 days leading up to their death.

While it is our opinion that the registered main diagnosis at discharge is a relevant proxy for urgency, our estimations will include patients who develop acute conditions during several days of hospitalization that are independent of the condition that led them into the ED.

When measuring our desired outcomes, we rely heavily on correct and timely registration of data and events, but we do not have the possibility to check the correctness of the data [20]. A significant amount of ED contacts were excluded due to missing data on diagnosis at discharge. To accommodate risk of bias, an additional analysis on the excluded cohort was completed (Additional file 1: Table S4, Additional file 1: Table S5).

Further our analyses are based on reliable triage in both systems, but we do not test the inter-rater agreement of either triage system.

### Interpretation

Clinicians in EDs that utilize triage systems based on vital signs only should be aware of the prognostic value of the presenting symptoms. Our data show that the vital signs of more than half of patients who experience adverse outcomes or diagnoses of critical illness, are normal or only slightly deviating at the time of triage. Standardized 5-level triage systems that include presenting symptoms carry higher prognostic value. Despite better prognostic performance, almost one in three of the patients admitted to an ICU within 24 h is triaged into a low-urgency category at ED arrival. Assessment of patients, when possible, should not be postponed because either triage system implies an immediate low-urgency condition. Early assessment and intervention might reduce the number of patients who experience adverse outcomes [33].

When implementing a triage system in an ED, the main objective of the selected system should be contemplated in relation to ED characteristics and available resources locally. A triage system based on vital signs only is better at identifying low-urgency patients, while a triage system including presenting symptoms will detect more critical conditions on the cost of systematic over-triage. Extensive over-triage can lead to inefficient use of staff and resources and is detrimental in situations of overcrowding in an ED [34].

## Conclusion

We find that a triage system based on vital signs and presenting symptoms is superior to a triage system using vital signs only. The high urgency categories of both triage systems are associated with adverse outcomes. DEPT achieves equal performance on the ability to detect ICU admission as other standardized 5-level triage systems while VITAL-TRIAGE is inferior. Future studies should investigate the performance of triage systems in subgroups of patients and identify potential weaknesses. Further, alternatives to vital signs and presenting complaints should be explored with the possibility to reach higher performance in triage systems.

## Supplementary Information

**Additional file 1.** Additional tables, analyses and triage algorithms.

## Data Availability

Not applicable.
