# Supplementary material for: Are 5-level triage systems improved by using a symptom based approach?—a Danish cohort study
Source: Scand J Trauma Resusc Emerg Med. 2022 Apr 25;30:31. doi: 10.1186/s13049-022-01016-2 (PMC9036764; doi:10.1186/s13049-022-01016-2)
Supplement: Supplementary file 1 — Additional file 1. Additional tables, analyses and triage algorithms. [file 13049_2022_1016_MOESM1_ESM.docx]

Supplementary files.

Supplementary Table 1
**Diagnoses of critical illness
Supplementary Table 1. Diagnoses of critical illness. ICD-10 codes.**

| **RKKP critical illness** | **ICD CODE** | **Name of diagnosis** |
| --- | --- | --- |
| **Stroke** | I60 *Excerpt I60.9 and i60.8 | subarachnoid hemorrhage |
|  | I61 | Intracerebral hemorrhage |
|  | I63 | Cerebral infarction |
|  | I64.9 | Stroke, not specified as hemorrhage or infarction |
|  | G45 *Excerpt G45.3 and G45.4 | Transient cerebral ischemic attacks and related syndromes |
| **Acute Coronary Syndrome** | I20.0 | Unstable angina pectoris |
|  | I21 | Acute myocardial infarction |
|  | I23 | Certain current complications following ST elevation (STEMI) and non-ST elevation (NSTEMI) myocardial infarction (within the 28-day period) |
|  | I24 | Other acute ischemic heart diseases |
| **Bleeding ulcer and gastrointestinal perforation.** | K22.3 | Perforation of esophagus |
|  | K25.0 | Acute gastric ulcer with hemorrhage |
|  | K25.1 | Gastric ulcer, Acute with perforation |
|  | K25.2 | Gastric ulcer, acute with both hemorrhage and perforation |
|  | K25.4 | Gastric ulcer, Chronic or unspecified with hemorrhage |
|  | K25.5 | Gastric ulcer, chronic or unspecified with perforation |
|  | K25.6 | Gastric ulcer, chronic or unspecified with both hemorrhage and perforation |
|  | K26.2 | Duodenal ulcer, acute with both hemorrhage and perforation |
|  | K26.4 | Duodenal ulcer, chronic or unspecified with hemorrhage |
|  | K26.5 | Duodenal ulcer, chronic or unspecified with perforation |
|  | K28.1 | Gastrojejunal ulcer, acute with perforation |
|  | K28.2 | Gastrojejunal ulcer, acute with both hemorrhage and perforation |
|  | K28.3 | Gastrojejunal ulcer, acute with hemorrhage or perforation |
|  | K28.5 | Gastrojejunal ulcer, chronic or unspecified with perforation |
|  | K28.7 | Gastrojejunal ulcer, chronic with hemorrhage or perforation |
|  | K27.0 | Peptic ulcer, site unspecified, Acute with hemorrhage |
|  | K27.1 | Peptic ulcer, site unspecified, Acute with perforation |
|  | K27.2 | Peptic ulcer, site unspecified, acute with both hemorrhage and perforation |
|  | K27,4 | Peptic ulcer chronic or unspecified with hemorrhage |
|  | K27.5 | Peptic ulcer, site unspecified, chronic or unspecified with perforation. |
|  | K27.6 | Peptic ulcer, site unspecified, chronic or unspecified with both hemorrhage and perforation |
|  | K35.2 | Acute appendicitis with generalized peritonitis |
|  | K57.0 | Diverticular disease of small intestine with perforation and abscess |
|  | K57.2B | Diverticular disease of large intestine with perforation and abscess |
|  | K57.4 | Diverticular disease of both small and large intestine with perforation and abscess |
|  | K62.8H | Other specified diseases of anus and rectum, non-traumatic perforation of rectum |
|  | K63.1 | Perforation of intestine (nontraumatic) |
|  | K82.2 | Perforation of gallbladder |
|  | K83.2 | Perforation of bile duct |

Supplementary Table 2

**Odds ratios for 24-hour ICU admittance
Stratified and unstratified odds ratios for admission to intensive care within 24 hours of ED arrival.**

| **Odds ratios 24-hour ICU admittance (stratified)** | | | | |
| --- | --- | --- | --- | --- |
| **variable** | **DEPT** | | **VITAL-TRIAGE** | |
|  | **Odds ratio** | **95% confidence interval** | **Odds ratio** | **95% confidence interval** |
| **Red** | 653.06 | 454.97 : 937.41 | 472.65 | 209.98 : 1063.89 |
| **Orange** | 60.78 | 42.32 : 87.31 | 136.36 | 60.37 : 308.01 |
| **Yellow** | 18.42 | 12.78 : 26.53 | 27.76 | 12.37 : 62.28 |
| **Green** | 4.72 | 3.22 : 6.92 | 8.42 | 3.74 : 18.96 |
| **Blue (ref)** | 1 | - | 1 | - |
| **Grey** | 39.36 | 27.4 : 56.51 | 18.68 | 8.32 : 41.93 |
|  | | | | |
| **Male** | 1.33 | 1.26 : 1.41 | 1.12 | 0.99 : 1.26 |
| **Age** | 1.01 | 1.00 : 1.01 | 1.01 | 1.01 : 1.01 |
|  | | | | |
| **CCI 1** | 1.42 | 1.32 : 1.54 | 1.42 | 1.20 : 1.68 |
| **CCI 2** | 1.39 | 1.26 : 1.52 | 1.62 | 1.34 : 1.95 |
| **CCI > 2** | 1.58 | 1.46 : 1.71 | 1.83 | 1.55 : 2.15 |
|  | | | | |
| **High urgency** | 15.37 | 14.33 : 16.48 | 17.80 | 15.79 : 20.05 |
| **Low urgency (ref)** | 1 | - | 1 | - |

| **Odds ratios 24-hour ICU admittance (unstratified)** | | | | |
| --- | --- | --- | --- | --- |
| **variable** | **DEPT** | | **VITAL-TRIAGE** | |
|  | **Odds ratio** | **95% confidence interval** | **Odds ratio** | **95% confidence interval** |
| **Red** | 879.04 | 612.79 : 1260.95 | 672,73 | 299.33 : 1511.92 |
| **Orange** | 84.55 | 58.91 : 121.33 | 212.36 | 94.22 : 478.62 |
| **yellow** | 24.59 | 17.08 : 35.41 | 43.34 | 19.36 : 97.04 |
| **Green** | 5.69 | 3.88 : 8.35 | 11.48 | 5.10 : 25.81 |
| **Blue (ref)** | 1 | - | 1 | - |
| **Grey** | 46.49 | 32.40 : 66.73 | 20.91 | 9.32 : 46.93 |
|  | | | | |
| **High Urgency** | 17.30 | 16.15 : 18.53 | 19.58 | 17.40 : 22.04 |
| **Low urgency (ref)** | 1 | - | 1 | - |

Supplementary Table 3
**Odds ratios for 24-hour ICU admittance
Stratified and unstratified odds ratios for two-day mortality.**

| **Odds ratios 2-day mortality (stratified)** | | | | |
| --- | --- | --- | --- | --- |
| **variable** | **DEPT** | | **VITAL-TRIAGE** | |
|  | **Odds ratio** | **95% confidence interval** | **Odds ratio** | **95% confidence interval** |
| **Red** | 51.11 | 40.30 : 64.83 | 14.59 | 10.47 : 20.32 |
| **Orange** | 5.79 | 4.56 : 7.35 | 3.21 | 2.25 : .58 |
| **Yellow** | 2.66 | 2.09 : 3.40 | 0.83 | 0.61 : 1.14 |
| **Green** | 0.99 | 0.75 : 1.29 | 0.36 | 0.26 : 0.49 |
| **Blue (ref)** | 1 | - | 1 | - |
| **Grey** | 11.07 | 8.77 : 13.96 | 1.08 | 0.79 : 1.49 |
|  | | | | |
| **Male** | 1.35 | 1.27 : 1.44 | 1.24 | 1.08 : 1.42 |
| **Age** | 1.06 | 1.05 : 1.06 | 1.06 | 1.06 : 1.07 |
|  | | | | |
| **CCI 1** | 1.16 | 1.06 : 1.28 | 1.42 | 1.16 : 1.74 |
| **CCI 2** | 1.48 | 1.35 : 1.63 | 1.64 | 1.33 : 2.03 |
| **CCI > 2** | 1.87 | 1.,72 : .2.03 | 2.00 | 1.67 : 2.40 |
|  | | | | |
| **High urgency** | 6.41 | 5.89 : 6.97 | 11.44 | 9.77 : 13.4 |
| **Low urgency (ref)** | 1 | - | 1 | - |

| **Odds ratios 2-day mortality (unstratified)** | | | | |
| --- | --- | --- | --- | --- |
| **variable** | **DEPT** | | **VITAL-TRIAGE** | |
|  | **Odds ratio** | **95% confidence interval** | **Odds ratio** | **95% confidence interval** |
| **Red** | 125.29 | 98.98 : 158.60 | 39.69 | 28.76 : 54.78 |
| **Orange** | 16.41 | 12.96 : 20.81 | 11.21 | 7.92 : 15.87 |
| **Yellow** | 6.77 | 5.32 : 8.61 | 2.87 | 2.11 : 3.91 |
| **Green** | 1.97 | 1.51 : 2.58 | 0.95 | 0.69 : 1.31 |
| **Blue (ref)** | 1 | - | 1 | - |
| **Grey** | 20.10 | 15.95 : 25.34 | 1.66 | 1.21 : 2.27 |
|  | | | | |
| **High Urgency** | 9.63 | 8.86 : 10.47 | 14.04 | 12.03 : 16.39 |
| **Low urgency (ref)** | 1 | - | 1 | - |

Supplementary Table 4
**Distribution of excluded patients into triage categories. The ED contacts were excluded due to missing data on diagnosis at discharge and length of stay.**

|  | **DEPT** | **VITAL-TRIAGE** |
| --- | --- | --- |
| **Triage categories** | | |
| **Grey** | 20,098 (65.1%) | 1,029 (35.7%) |
| **Blue** | 423 (1.4%) | 122 (4.2%) |
| **Green** | 1,498 (4.9%) | 1,128 (39.2%) |
| **Yellow** | 3,317 (10.8%) | 517 (18.0%) |
| **Orange** | 4,028 (13.1%) | 49 (1.7%) |
| **Red** | 1,488 (4.8%) | 35 (1.2%) |

Supplementary Table 5
 **The table shows the distribution of excluded ED contacts into 24-hour ICU admission, 2-day mortality and 48-hour surgery. Presented by triage category vertically and the triage system horizontally. The ED contacts were excluded due to missing data on diagnosis at discharge and length of stay.**

| **Triage category** | **DEPT** | **VITAL-TRIAGE** |
| --- | --- | --- |
| **24-hour ICU** | | |
| **Grey** | 0 | 0 |
| **Blue** | 0 | 0 |
| **Green** | 0 | 0 |
| **Yellow** | 0 | 0 |
| **Orange** | 0 | 0 |
| **Red** | 0 | 0 |
| **2-day mortality** | | |
| **Grey** | 765 | 25 |
| **Blue** | 15 | 1 |
| **Green** | 12 | 6 |
| **Yellow** | 25 | 10 |
| **Orange** | 43 | 2 |
| **Red** | 49 | 8 |
| **48-hour surgery** | | |
| **Grey** | 0 | 0 |
| **Blue** | 0 | 0 |
| **Green** | 0 | 0 |
| **Yellow** | 0 | 0 |
| **Orange** | 0 | 0 |
| **Red** | 0 | 0 |

|  |  |  |  |  |  |
| --- | --- | --- | --- | --- | --- |
|  | **1 Red** | **2 Orange** | **3** **yellow** | **4 Green** | **5 Blue** |
|  | **Lifethreatening** | **urgent** | **Less urgent** | **Non-urgent** | **Minor injuries** |
| **A** |  |  |  |  |  |
| **B** | **SpO_2_ < 90% with 0_2_** | **SpO_2_ < 95% with 0_2_** | **SpO_2_ ≥ 95% with 0_2_** | **SpO_2_ ≥ 95 % without 0_2_** |  |
|  | **SpO_2_ < 80% without 0_2_** | **SpO_2_ < 90% without 0_2_** | **RF > 25** | **RF 12 – 25** |  |
|  | **RF > 35 or < 8** | **RF > 30** |  |  |  |
| **C** | **HR > 140** | **HR > 120 or < 40** | **HR > 110 eller < 50** | **HR 50 – 110** |  |
|  | **BP_sys_ < 80 mmHg** | **BP_sys_ < 90 mmHg** |  | **BT_sys_ ≥ 90 mmHg** |  |
| **D** |  |  | **VAS 4-10** | **VAS 4-10** | **VAS 0-3** |
|  | **GCS ≤ 8** | **GCS: 9 - 13** | **GCS = 14** | **GCS = 15** |  |
| **E** | **Tp < 32^0^ C** | **Tp > 40^0^ C**  **Tp: 32 - 34^0^ C** | **Tp > 38^0^ C** | **Tp: 35 - 38^0^ C** |  |
|  |  |  |  |  |  |
|  |  | | | |  |

Supplementary Table 6
 **The table shows the vital sign triage algorithm used by DEPT and VITAL-TRIAGE.**

Supplementary Table 7 **The table shows the presenting symptom algorithm utilised by DEPT in case of patients with chest pain**

|  |  |  |  |  |  |
| --- | --- | --- | --- | --- | --- |
|  | **1 Red** | **2 Orange** | **3** **yellow** | **4 Green** | **5 Blue** |
|  | **Life threatening** | **urgent** | **Less urgent** | **Non-urgent** | **Minor injuries** |
| **Squeezing/pressing/pinching pain or discomfort** |  | **Yes, during the last day** |  |  |  |
| **Duration** |  | **Minutes to hours -persistent** | **Minutes - transient** |  |  |
|  |  |  |  |  |  |
|  |  |  |  |  |  |
| **ECG abnormalities** |  | **New ST-T changes  or wide complex arrythmia** | **Small complex arrythmia** | **None** |  |
|  |  |  |  |  |  |
|  |  |  |  |  |  |

|  |  |  |  |  |  |
| --- | --- | --- | --- | --- | --- |
|  | **1 Red** | **2 Orange** | **3** **yellow** | **4 Green** | **5 Blue** |
|  | **Life threatening** | **urgent** | **Less urgent** | **Non-urgent** | **Minor injuries** |
| **Suspicion of upper airway obstruction** | **Stridor or not able to swallow saliva** | **Swelling of neck/throat** |  |  |  |
| **Level of dyspnoe and exhaustion** | **Difficulty breathing while at rest and/or**  **Respiratory exhaustion** | **Newly arrived or worsening of habitual dyspnoea at rest** | **Newly arrived dyspnoea at exertion** | **Light dyspnoea at exertion** |  |
|  |  |  |  |  |  |
|  |  |  |  |  |  |
| **Suspicion of airway damage** | **Fire or corrosive damage or penetrating trauma against head/neck** | **Trauma against neck or thorax.** |  |  |  |
|  |  |  |  |  |  |
| **Cough** |  | **Multiple haemoptysis last 24 hours** | **Purulent expectorate or single episode of haemoptysis** | **Cough without expectorate** |  |
| **Pain while breathing** |  | **VAS 4-10**  **including at least one of:**  **- yellow vital sign score - pain affected movement pattern  - affected general condition** | **VAS 4-10  Including at least one of:**  **- green vital sign score - uninhibited movement pattern  - unaffected general condition** | **VAS 0-3 including at least one of:  - green vital sign score - uninhibited movement pattern  - unaffected general condition** |  |
| **I.V/I.m opioids before 120 minutes after arrival** |  | **Yes and yellow vital sign score** | **Yes and green vital sign score** |  |  |
| **ECG** |  | **New ST-T changes or wide complex arrhythmia** | **Small complex arrhythmia** |  |  |

Supplementary Table 8
**The table shows the presenting symptom algorithm utilised by DEPT in case of patients with symptoms from the airways.**
